# Effects of high-intensity interval training on blood lactate levels and cognition in healthy adults: protocol for systematic review and network meta-analyses

**DOI:** 10.1186/s13643-021-01874-4

**Published:** 2022-02-19

**Authors:** Nithin Jacob, Isis So, Bhanu Sharma, Susan Marzolini, Maria Carmela Tartaglia, Robin Green

**Affiliations:** 1 KITE Research Institute, Toronto Rehabilitation Institute-University Health Network, Toronto, Ontario Canada; 2 Rehabilitation Sciences Institute, University of Toronto, Toronto, Ontario Canada; 3 Institute of Medical Science, University of Toronto, Toronto, Ontario Canada; 4 Department of Medical Sciences, McMaster University, Toronto, Ontario Canada; 5 Tanz Centre for Research in Neurodegenerative Diseases, University of Toronto, Kembril Research Institute, Toronto Western-University Health Network, Toronto, Ontario Canada; 6 Department of Psychiatry, Division of Neurosciences and Clinical Translation, University of Toronto, Toronto, Ontario Canada

**Keywords:** Blood lactate, High-intensity interval training, Cognition, Exercise

## Abstract

**Background:**

High-intensity interval training (HIIT) has shown to confer cognitive benefits in healthy adults, via a mechanism purportedly driven by the exercise metabolite lactate. However, our understanding of the exercise parameters (e.g., work interval duration, session volume, work-to-rest ratio) that evoke a peak blood lactate response in healthy adults is limited. Moreover, evidence relating HIIT-induced blood lactate and cognitive performance has yet to be reviewed and analyzed. The primary objective of this systematic review is to use network meta-analyses to compare the relative impact of different HIIT work-interval durations, session volumes, and work-to-rest ratios on post-exercise blood lactate response in healthy adults. The secondary objective is to determine the relationship between HIIT-induced blood lactate and acute post-HIIT cognitive performance.

**Methods:**

A systematic review is being conducted to identify studies measuring blood lactate response following one session of HIIT in healthy adults. The search was carried out in (1) MEDLINE, (2) EMBASE, (3) Cochrane Central Register of Controlled Trials, (4) Sport Discus, and (5) Cumulative Index to Nursing and Allied Health Literature Plus with Full Text (CINAHL+). After abstract and full-text screening, two reviewers will independently extract data on key outcomes variables and complete risk of bias assessment using the Cochrane Risk of Bias Tool and the Risk of Bias in Non-Randomized Studies of Interventions tool. Network meta-analyses will be used to generate estimates of the comparative effectiveness of blood lactate on cognitive outcomes using corresponding rankings for each work-interval duration, session volume, and work-to-rest ratio category. Where applicable, meta-regressions analyses will be performed to test the relationship between changes in the blood lactate and changes in cognitive performance. Analyses will be conducted using MetaInsight Software.

**Discussion:**

This study will provide evidence on how to structure a HIIT protocol to elicit peak blood lactate response in healthy adults and will increase our understanding of the relationship between HIIT-induced blood lactate response and associated cognitive benefits.

**Systematic review registration:**

PROSPERO CRD42020204400

## Background

High-intensity interval training (HIIT) is a growing fitness trend [58]. This type of training is characterized by repeated short-to-long bouts of intense exercise (i.e., ≥ 80% of peak or maximum heart rate [64]) separated by intervals of recovery or rest [5]. HIIT is an alternative to moderate-intensity continuous training (i.e., traditional endurance training) with similar adherence rates [57], yet the former may result in greater improvements to cardiorespiratory fitness in young, middle-aged, and older adults [37, 44, 65], and greater enjoyment of exercise protocols in healthy young adults [22, 59] with reduced time commitment [19]. Insufficient time has been cited as a main constraint to exercise in young, middle aged, and older adults [7, 15, 60].

In addition to other health benefits, HIIT has been demonstrated to result in cognitive benefits in healthy adults, even after a single session [30]. A recent systematic review [25] has shown that one session of HIIT has positive acute effects on executive function in healthy adults. These single bouts of exercise are purported to improve executive function by increasing circulating levels of peripheral exercise factors, including lactate [25].

Lactate is an essential biomarker for neuronal metabolism and excitability [33], a marker of metabolic stress [34], and importantly it is a neuronal energy source alternative to glucose [45]. Studies have indicated that lactate derived from the skeletal muscle can be taken up and metabolized by the brain [9, 10, 12].

Following HIIT, there is increased neuronal activation in the prefrontal cortex [28, 29, 54]. Neurons can use lactate to meet these heightened energy demands [31], and thus, the exercise metabolite plays a role in the enhanced executive functioning that is observed [30, 31].

Lactate also serves as a signaling molecule within neurons to increase the expression of brain-derived neurotrophic factor (BDNF) [39]. BDNF is a protein produced in both the central [46] and peripheral [32] nervous system, but 75% of the neurotrophin originates from the brain [46]. This protein, upregulated by exercise [2, 13, 16, 53], supports neurogenesis, neuronal survival, synaptic plasticity, and dendritic spine growth [36], and plays a key role in learning and memory [62]. In healthy adults, increased peripheral blood lactate has been associated with circulating BDNF [17, 47, 52]. Exercise sessions eliciting peak blood lactate accumulation may consequently increase BDNF expression in the brain [17, 31], as measured through increased peripheral BDNF levels [42]. Given that BDNF is an important candidate behind exercise-induced neuronal plasticity and learning enhancement [20], acute increases in BDNF may give rise to enduring cognitive and neural benefits.

To date, data on the relationship between HIIT protocols parameters and the ensuing acute blood lactate response has yet to be consolidated in the literature. This understanding is important because a HIIT protocol eliciting peak blood lactate accumulation may in turn increase availability of lactate as an energy substrate for neurons—which may in turn increase the transient gains in executive function following exercise.

The effect of HIIT on blood lactate is influenced by exercise protocol parameters such as work interval duration, intensity, and session volume [1, 8]. However, HIIT protocols studied to date in humans vary considerably with respect to these parameters, which currently makes it difficult to draw conclusions on what type of HIIT protocol best promotes blood lactate and its downstream physiological, cognitive, and neural effects in healthy adults. A systematic review of the literature that compares different HIIT protocols (including sprint-interval training; SIT) with respect to the effect on the blood lactate is therefore needed. To that end, the proposed systematic review and network meta-analyses will address the following research questions:

1. Individually, what is the work-interval duration, session volume, and work-to-rest ratio that is most effective, if at all, in increasing blood lactate levels immediately following HIIT/SIT in healthy adults?
2. What is the relationship, if any, between HIIT/SIT-induced blood lactate levels and acute post-exercise cognitive performance in healthy adults?

The results will provide researchers and exercise professionals with a clearer understanding of how to optimally prescribe HIIT/SIT session protocols to elicit peak peripheral blood lactate concentrations. The results will also advance our understanding of the impact of such increases on cognitive functioning, specifically executive functioning, learning, and memory.

## Methods

This protocol follows the Preferred Reporting Items for Systematic Reviews Involving a Network Meta-Analysis (PRIMSA-NMA) [26]. This review has been registered in the PROSPERO database (CRD42020204400).

### Eligibility criteria

A priori inclusion and exclusion criteria will be used to evaluate study eligibility under the PICOS framework.

### Population

We will include studies with participants that are healthy and aged 18 years or older. Healthy participants are defined as individuals who are not described as having been hospitalized or diagnosed with a disease/dysfunction and/or receiving medical treatment for a disease [50] at the time of the study. We will not include studies in pregnant people or institutionalized adults. Study arms will be excluded if any of its participants have documented medical comorbidities. Healthy control arms in studies examining the effects of exercise on a disease group will be included if data are stratified by cohort and the healthy control data can be analyzed separately.

### Intervention

This review will exclude studies that only investigate resistance training or moderate continuous exercise. We will include studies that measure blood lactate following one session of high-intensity/sprint interval training. High-intensity interval training (HIIT) is an exercise protocol consisting of high-intensity work intervals (≥ 80% of maximum or peak heart rate), followed by low-intensity recovery intervals [64]. Sprint interval training (SIT) is an intermittent exercise protocol involving workloads that exceed the normal requirement to reach peak oxygen consumption (i.e., maximal all-out effort) [19, 49].

Number of exposures: Studies evaluating blood lactate levels after one full session of HIIT or SIT will be included. (N.b., a session of HIIT/SIT can be comprised of multiple, individual intervals of high-intensity exercise). If multiple sessions of a HIIT/SIT protocol are completed on a single day, then only information regarding the very first session will be used in the analyses. This is because repeated HIIT/SIT lowers the blood lactate response [61].

Exposure variance: Included studies must have the HIIT/SIT as the first exercise session of the day. Studies will be excluded if participants engage in other exercise interventions (e.g., resistance training, circuit training) prior to HIIT/SIT on the same day. Only information regarding the HIIT/SIT will be used in the analyses if the data can be stratified. The recovery interval that follows the work interval can be passive (complete rest) or active (low-intensity effort) recovery.

Exposure modality: Studies that administer HIIT/SIT through a standardized, replicable protocol will be included, such as—but not exclusive to—swimming, cycling, running, or treadmill. HIIT/SIT administered through sporting games (e.g., soccer, judo) will be excluded since the stimulus cannot be precisely replicated between subjects. HIIT/SIT involving only upper body (e.g., arm ergometer) will be excluded since the work rate max and VO_2 _peak achieved is lower during maximal upper body exercise compared to lower body exercise (e.g., cycle ergometer) [66].

Exercise protocol parameters to be collected include frequency (i.e., number of work/recovery intervals), intensity (i.e., percentage of peak heart rate or power output), modality (e.g., upright cycle ergometer), work/recovery interval duration, session volume (i.e., total exercise time without warm-up or cool-down), and heart rate levels.

### Study type

All full-text, peer-reviewed primary research will be included that involved healthy adults and aimed to determine the acute effects of a high-intensity interval protocol on blood lactate levels. We expect that most study designs will be observational, examining the impact of a stimulus (i.e., high-intensity interval training) on the outcome of interest (i.e., blood lactate). Longitudinal studies will only be included if the blood lactate was measured immediately after the first session of HIIT/SIT. A subset of these studies will also have measured cognitive performance following a session of HIIT/SIT. Qualitative studies, study protocols without a published study, gray literature, and published abstracts will be not be included in this review. The authors acknowledge that the exclusion of these types of studies may increase the chances of publication bias in this systematic review.

### Outcomes

Primary outcome: Studies must measure blood lactate (mmol/L) immediately after one session of HIIT/SIT through the whole blood, serum, or plasma samples, and not the muscle lactate or saliva. Examples of how blood samples can be procured include—but are not exclusive to—the finger prick, ear lobe prick, and blood draws from the limbs. Blood lactate levels must be analyzed immediately after procurement of blood samples, or samples must be cold stored, stabilized, and sent off to a laboratory for analysis at a later time using a lactate analyzer.

Secondary outcome: A subset of included studies will have measured cognitive performance pre-and post-acute HIIT/SIT. Cognitive assessments can be for executive function (e.g., working memory, inhibitory control, set shifting) and/or for learning and memory (e.g., paired associate learning, story recall, word-list learning and recall, visuospatial learning and delayed recall).

### Information sources

The search strategy includes main keywords of “high-intensity interval training” and “blood lactate.” The full search strategy for this review can be found as an additional file. The search strategy was pilot tested in MEDLINE and developed in collaboration with a health sciences librarian.

The search was carried out by the first reviewer in the following five databases: (1) MEDLINE, (2) EMBASE, (3) Cochrane Central Register of Controlled Trials, (4) Sport Discus, and (5) Cumulative Index to Nursing and Allied Health Literature Plus with Full Text (CINAHL+). In addition to electronic database searches, we will cross-reference the reference lists of included studies for potential articles that meet the inclusion criteria, check the articles citing those that are included in the review to see if they are eligible for inclusion, and check the e-pub ahead of print section of journals in the field for any additional relevant articles.

Date last searched: August 21, 2020

Language restrictions: English language only

Search

**Table:** 

**MEDLINE search strategy**	
**MeSH Term for intervention** 1. Exp high-Intensity Interval Training/	
**Text word for intervention** 2. ((High adj2 Intensity) adj2 Interval*).tw,kf 3. ((High adj2 Intensity) adj2 Aerobic*).tw,kf 4. ((High adj3 Intensity) adj3 (Exercis*)).tw,kf 5. ((High adj2 Intensity) adj2 (Intermit*)).tw,kf 6. ((High adj2 Intensity) adj2 (Training*)).tw,kf 7. ((High adj2 Intensity) adj2 (Circuit*)).tw,kf 8. ((High adj2 Intensity) adj2 Workout*).tw,kf 9. ((High adj2 Intensity) adj2 Cycl*).tw,kf 10. ((High adj2 Intensity) adj2 (repetition or repeat*)).tw,kf 11. Interval Exercis*.tw,kf 12. Interval training*.tw,kf 13. (Intermit* adj2 ((exercis*) or (training*))).tw,kf 14. Sprint*.tw,kf 15. HIIT.tw,kf 16. HIIE.tw,kf 17. SIT.tw,kf 18. Or/1-17 [**HIIT]	
**MeSH Term for outcomes** 19. Exp lactates/ or exp lactic acid/	
**Text word searches for outcomes** 20. Lactate.tw,kf 21. ((Blood or plasma or serum) adj2 lactic).tw,kf 22. Or/19-21	
23. 18 and 22 24. 23 not (exp animals/ not exp humans/)	

### Study selection

Results of the full-search strategy will be imported into Clarivate Analytics Endnote X9.3.3 (2020) wherein duplicates will be removed. The de-duplicated search results will be imported into Covidence. Before abstract screening, the third reviewer (BS) will create a training set comprised of randomly selected articles (only titles and abstracts, ~2% of the entire sample). The first two reviewers (NJ, IS) will independently practice title/abstract screening using an eligibility criteria guidance document. Disagreements will be resolved through discussions with the third reviewer and the guidance document will be updated to improve clarity of eligibility criteria. Another similar training set will be completed before the full-text screening stage using randomly selected full-text articles.

After training, the two independent reviewers will evaluate the title and abstracts of all articles for classification as eligible, maybe, or non-eligible in Covidence. Full-text review will be completed by the two independent reviewers to determine the final studies to be included. Excluded full-text articles will be compiled in Covidence with their respective reasons for exclusion (e.g., design, population, protocol, outcome, etc.).

Discrepancies between the first two reviewers at the abstract and full-text screening stage will be resolved through discussions with the third reviewer and reference to the a priori inclusion/exclusion criteria.

Performing valid indirect comparisons in network-meta analyses requires that the different sets of randomized trials are similar on important factors other than the intervention comparison being made. This requirement is termed transitivity [6]. Inclusion of studies in the network-meta-analyses will be determined after data extraction has occurred and will be dependent on whether the study data violates transitivity.

### Data collection process and data items

Data extraction will be completed by the two reviewers responsible for the screening phase using a customized extraction worksheet in Excel that is finalized prior to full-text screening. The worksheet will be piloted on ~5% of the included studies, and changes to the form will be made as needed and documented. The two authors will independently extract the following information: author and study information, participant information, exercise protocols, blood lactate, and cognitive performance outcomes. We will make all efforts to contact the authors to gather any relevant missing data. If we are not successful in obtaining the missing information, then the specific study will be excluded from analyses. Table 1 below shows all the variables we plan to extract from the included full-texts.

**Table 1:** Variables to be extracted at the full-text stage

Bibliographic	• Authors • Title • Year of publication • Journal • Sources of funding • Institutions and affiliations	• Reported conflict of interest • Study design • Country • Setting(s) • Type of allocation sequence • Inclusion and exclusion criteria outlined by the study
Demographics	• Age • Sex • Sample size	• Description of health status • Physical activity levels • Fitness levels
Exercise protocol	• Warm-up and cool-down duration, intensity, and modality • Exercise modality (e.g., treadmill, stationary cycle) • Number and duration of work/recovery intervals	• Exercise intensity • Exercise session volume (total time of work and recovery intervals)
Blood lactate outcomes	• Time difference between exercise termination and blood procurement • Method of blood draw (e.g., finger prick, earlobe prick, or blood draw from limbs)	• Baseline and post-exercise blood lactate levels (mmol/L) with standard deviations
Cognitive performance outcomes	• Time difference between exercise termination and cognitive assessment • Cognitive tests and domain	• Baseline and post-exercise cognitive scores with standard deviations
Additional variables	• Peak oxygen uptake peak heart rate, and work rate from graded exercise test • Average heart rate, work rate, and oxygen uptake during HIIT/SIT	• Adverse effects (e.g., reasons for dropout, dizziness) • Recruitment, retention, adherence, outcome rates and acceptability of intervention

### Classification of experimental arms

Classification of the arms will be carried out at the data extraction stage. HIIT/SIT protocols of each study will be grouped into categories based on different work-interval durations, session volumes, and work-to-rest ratios. Network-meta analyses will be performed to evaluate the effect of these different categories on blood lactate levels. Work-interval duration is defined as the period of the exercise protocol where intensity is greater or equal to 80% of heart rate maximum or peak. Session volume is defined as the total time duration of the work and recovery intervals, excluding warm-up and cool-down. Work-to-rest ratio is defined as the work-interval duration divided by the recovery-interval duration. Any disagreements regarding categorization will be resolved through discussions with the third reviewer.

The following work-interval duration categories will be used:

**Table:** 

≤ 10s	>10 and ≤30s		
≤ 30s (short interval)		>30s and <2min (medium interval)	≥2 min (long interval)

The following session volume categories will be used:

**Table:** 

≤ 5 mins (low volume)	> 5min and < 15 min (medium volume)	≥15 min (high volume)

The following work-to-rest ratio categories will be used:

**Table:** 

1:1 (e.g., 30-s work; 30-s recovery)	1:2 and 2:1 (e.g., 30-s work, 60-s recovery)	1:3 and 3:1 (e.g., 10-s work, 30-s recovery)

### Geometry of the network

We will depict the number of articles from which the information comes (treatment nodes) in a network plot. Direct, indirect, or mixed comparisons and the number of participants with different comparisons will be shown using the size of nodes. The width of the line between interventions will show the number of trials included in each comparison. The geometric shape will be based on the conditions that were studied. For example, if all the interventions are compared with placebo, the geometry will be that of a star. On the other hand, if all the interventions are compared with other HIIT protocols, the geometry will be in a polygon form [38].

### Risk of bias within individual studies

For interventions employing randomization procedures, the risk of bias for each main outcome will be assessed independently by two researchers using version 2 of the Cochrane Risk of Bias Tool [23, 24]. The criteria are as follows: (1) randomization process, (2) deviations from intended interventions, (3) missing outcome data, (4) measurement of outcomes, and (5) selection of the reported results. The funnel plot and the Egger test [14] will be used to examine publication bias [23, 24]. For non-randomized interventions, we will use the Risk of Bias in Non-Randomized Studies of Interventions (ROBIN-I) tool [56]. This tool uses “signalling questions” to judge the risk of bias within seven distinct domains. The judgements within each domain carry forward to an overall risk of bias judgement for each main outcome [55]. Two independent reviewers will assess each study, and disagreements will be discussed and resolved through the third reviewer.

### Quality assessment for exercise prescription

There are many variables that can be manipulated when prescribing HIIT/SIT which include the exercise modality, the number, duration, and intensity of the work and recovery intervals [5].

In order to assess the quality of the HIIT protocol prescription, we will consider the following questions, which are informed from the American College of Sports Medicine 110th edition [48].

1. Does the HIIT session have a warm-up, cool-down, and stretching?
2. Is the intensity prescribed based on an incremental (graded) exercise test (e.g., cardiopulmonary exercise test, symptom limited test)?
3. Is heart rate *reserve*, VO_2_ *reserve*, ventilatory threshold, or respiratory compensation point used for estimating exercise intensity? The exercise intensity can be underestimated or overestimated when using percentage of heart rate *max* or VO_2_ *max*.
4. Is the intensity quantitatively measured (e.g., heart rate, power output, absolute oxygen uptake, MET) during the HIIT protocol?
5. Is the HIIT modality (e.g., stationary cycling) the same as the one used for the exercise test?
6. Do participants receive a familiarization period for the HIIT protocol? For example, do they come to the laboratory at a separate time to perform a version of the HIIT protocol, so they are accustomed to the repeated efforts and the equipment?

The above are all variables that we plan to include as logistic covariates into regression models and use for stratifying the results by quality level. We do not plan to exclude studies based on the above quality assessment questions.

### Summary measures

Network meta-analyses produces a set of network estimates of the intervention effects for all basic comparisons. These intervention effects (measured as mean differences) will be calculated for blood lactate levels between all the categories for work-interval duration, session volume, and work-to-rest ratio.

The summary measures will be mean differences between treatment arms (with 95% confidence intervals). The mean difference will be obtained by subtracting the blood lactate levels post-exercise from the blood lactate levels pre-exercise. If a study does not provide standard deviations for blood lactate measurements, or it is not possible to calculate, the study will be excluded from analyses. To interpret the comparative effectiveness of all nodes in the network, the data will be summarized using treatment rankings and a surface under the cumulative ranking curve.

We will show the distribution of ranking probabilities for each treatment arm by drawing probability lines, which are known as rankograms [51].

### Planned method of analysis

#### Assessment of transitivity

We will compile a table of study characteristics that may act as effect modifiers to assess the level of transitivity. Potential effect modifiers include age, sex, ethnicity, exercise modality, sequence order of HIIT/SIT and other exercise training, and effect of measure (e.g., mean difference, odds ratio, relative risk) for blood lactate levels and cognitive assessments.

### Pairwise meta-analysis and meta-regression

We expect only a small subset of the extracted data to be eligible for network meta-analyses due to the requirement of within subject comparisons. Thus, we plan to analyze the remaining studies using categorical meta-analyses (RevMan5.4) and meta-regression (Rstudio).

For both blood lactate and cognitive outcomes, we will conduct exploratory pairwise meta-analyses and meta-regression using a random, or mixed-effect (whichever is appropriate) model, and generate forest plots with individual and pooled effect sizes. Where possible, the aforementioned categories for work-interval duration (e.g., small ≤30), session volume (e.g., low ≤ 5min), and work-to-rest ratios (e.g., 1:1) will be used for the categorical meta-analyses and meta-regression. The results from the categorical meta-analyses and meta-regression will be presented as forest plots and regression models, respectively. We will use the funnel plot and the Egger test to examine publication bias [3].

### Network meta-analyses

If the transitivity assumption is met, random-effects network meta-analyses will be conducted within a Frequentist framework [11]. Pairwise comparison estimates for each category will be in tabular format in the final manuscript, and rankings will represent the probability of each node producing the best outcome. The rankings will be presented with mean ranks, 95% confidence intervals, and the surface under the cumulative ranking curve. We will assess the convergence by evaluating the trace plots and convergence criteria [4].

#### Assessment of inconsistency, heterogeneity, and quality of the evidence

We will use the chi-square test and the *I*^2^ statistic to assess the percentage of variability across studies attributable to heterogeneity. The random effects model will be used if the heterogeneity test shows statistical significance (*I*^2^ >50%, *p*<0.05). Otherwise, we will adopt a fixed-effects model. If the number of included studies in the meta-analyses is not enough (<10), potential publication bias will not be assessed. We will adjust the number of subjects for clinical trials that have more than two arms when we perform network meta-analyses.

We will consider design inconsistency and loop inconsistency since the included studies will likely consist of a mixture of two-arm and multi-arm studies. This will be achieved by applying a design-by-treatment interaction model. If inconsistency is indicated in the network, any closed loops within the network will be assessed [27]. If the transitivity assumption is not testable, we will report consistency statistics as an index of the transitivity assumptions for closed loops.

#### Risk of bias across studies

We plan to use the CINeMA (Confidence in Network Meta Analysis) tool [41, 43] to assess the quality of the evidence across included studies. The CINeMA considers 6 domains: within study bias, reporting bias, indirectness, imprecision, heterogeneity, and incoherence, and assigns judgements at 3 levels (no concerns, some concerns, or major concerns). We will determine effect levels of confidence for each treatment corresponding to the GRADE assessments of very low, low, moderate, or high.

#### Additional analyses

Subgroup analyses will be performed, for example, on studies involving (1) participant fitness, (2) only older adults (i.e., ages 65 and over), (3) work interval intensities that are ≥90% heart maximum or peak, (4) plasma lactate vs whole blood lactate, (5) venous vs arterialized blood samples, (6) cycle vs treadmill HIIT modality, (7) and sex differences. If studies are primarily conducted within one sex, we plan to analyze the male vs female data separately in the regression models, where possible.

Sensitivity analyses will be performed after removing studies classified as having a high risk of methodological bias.

## Discussion

Research interest in HIIT is growing rapidly [35]. This time-efficient exercise regime [40] purportedly improves cardiovascular and metabolic fitness in healthy adults [37] and patients [64]. A growing evidence base is now also pointing to the benefits of HIIT on cognitive outcomes [18, 25]. However, there are numerous variations of HIIT being studied, making it difficult to determine which protocol is most suitable to achieve a specific goal in a given population. To address this gap, Wen and colleagues [63] determined the work interval durations, session volumes, and training periods that are most effective for improving maximal oxygen uptake in the general population. They concluded that long-intervals (≥2 mins), high-volume (≥15min), and moderate to long-term (≥4–12 weeks) HIIT protocols maximize the training effects on maximal oxygen uptake. Likewise, we will assess similar HIIT parameters to determine the regimen most effective in eliciting metabolic stress (i.e., blood lactate accumulation) in healthy adults. Exercise metabolites such as lactate are purported to play a role in enhancing cognitive performance following HIIT in humans [21, 31, 61]. To ensure or more greatly enhance the effects of HIIT on cognition, it is important to determine the optimal HIIT protocol parameters that result in peak blood lactate concentrations.

No previous systematic review has determined the optimal HIIT protocol parameters to increase blood lactate levels. Findings from the current network meta-analyses will guide researchers on the design and prescription of HIIT protocols that generate maximal metabolic stress in healthy adults. The secondary examinations of the relationship between HIIT/SIT, blood lactate, and cognitive outcomes will inform future exercise research in the area of cognition in clinical populations.

## Data Availability

This protocol manuscript does not contain any data. Data extraction has not yet started. All data generated or analyzed during this review will be included in the published article.

## Abbreviations

BDNF: Brain-derived neurotrophic factor
HIIT: High-intensity interval training
SIT: Sprint-interval training
